# Supplementary material for: 3D Modeling of Silver Doped ZrO2 Coupled Graphene-Based Mesoporous Silica Quaternary Nanocomposite for a Nonenzymatic Glucose Sensing Effects
Source: Nanomaterials (Basel). 2022 Jan 7;12(2):193. doi: 10.3390/nano12020193 (PMC8779333; doi:10.3390/nano12020193)
Supplement: Supplementary file 1 [file nanomaterials-12-00193-s001.zip › nanomaterials-1520747-supplementary.pdf]

Supplementary Materials

3D Modeling of Silver Doped ZrO_2 Coupled Graphene-Based Mesoporous Silica Quaternary Nanocomposite for a Nonenzymatic Glucose Sensing Effects

Kamrun Nahar Fatema [1], Chang-Sung Lim [1], Yin Liu [2], Kwang-Youn Cho [3], Chong-Hun Jung [4] and Won-Chun Oh [1,2,*]

1 Department of Advanced Materials Science & Engineering, Hanseo University, Seosan-si 356-706, Chungnam, Korea; kamrunnahar270@gmail.com (K.N.F.); cslim@hanseo.ac.kr (C.S.-L.)
2 Anhui International Joint Research Center for Nano Carbon-Based Materials and Environmental Health, College of Materials Science and Engineering, Anhui University of Science & Technology, Huainan 232001, China; yinliu@aust.edu.cn
3 Korea Institutes of Ceramic Engineering and Technology, Soho-ro, Jinju-si 52851, Gyeongsangnam-do, Korea; kycho@kicet.re.kr
4 Decommissioning Technology Research Division, Korea Atomic Energy Research Institute, P.O. Box 105, Yuseong, Daejeon 305-600, Korea; nchjung@kaeri.re.kr
* Correspondence: wc_oh@hanseo.ac.kr; Tel.: +82-41-660-1337; Fax: +82-41-688-3352

(a)

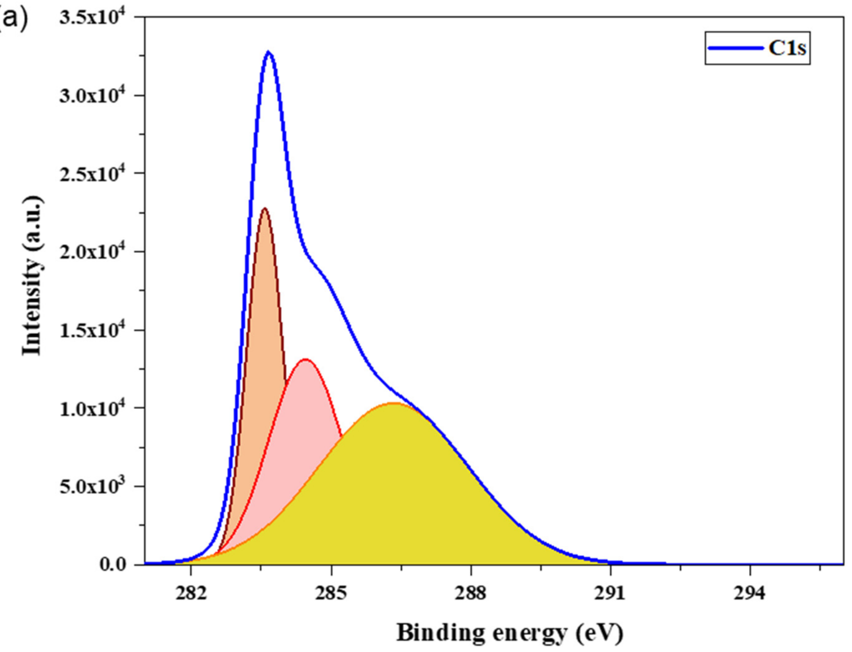

C1s
3.5x10^4
3.0x10^4
2.5x10^4
2.0x10^4
1.5x10^4
1.0x10^4
5.0x10^3
0.0
Intensity (a.u.)
282
285
288
291
294
Binding energy (eV)

(b)

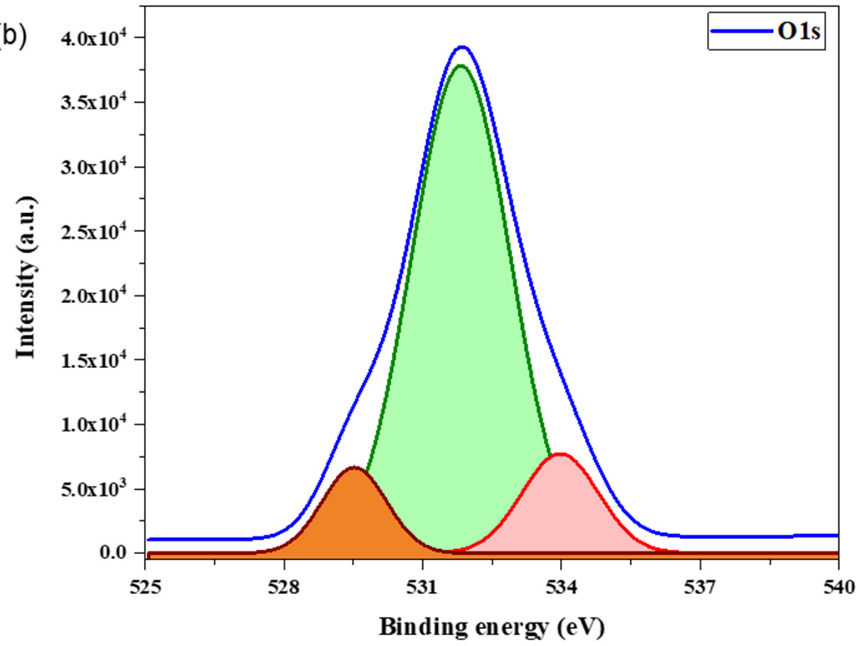

O1s
4.0x10^4
3.5x10^4
3.0x10^4
2.5x10^4
2.0x10^4
1.5x10^4
1.0x10^4
5.0x10^3
0.0
Intensity (a.u.)
525
528
531
534
537
540
Binding energy (eV)

(c)

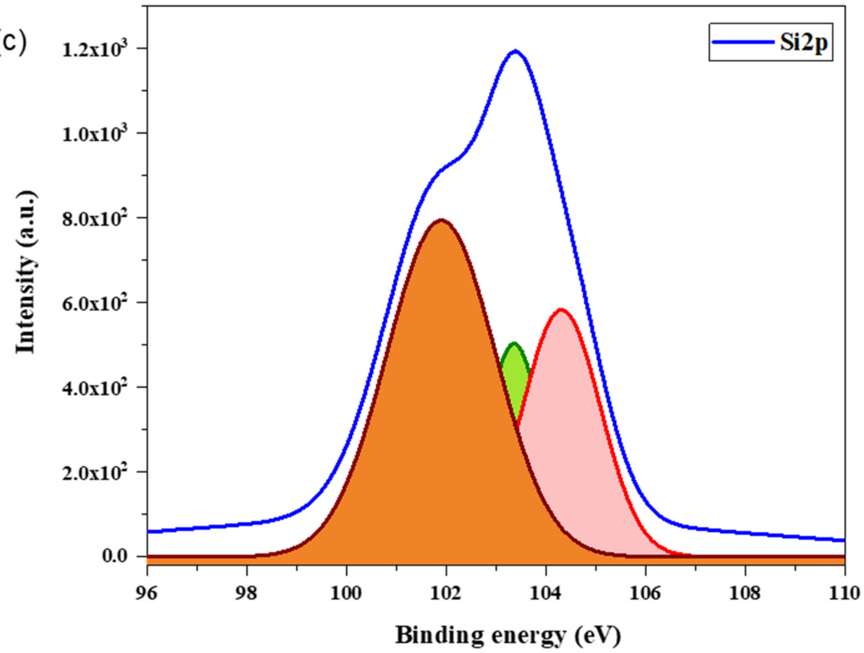
Si2p
Intensity (a.u.)
1.2x10^3
1.0x10^3
8.0x10^2
6.0x10^2
4.0x10^2
2.0x10^2
0.0
96
98
100
102
104
106
108
110
Binding energy (eV)

(d)

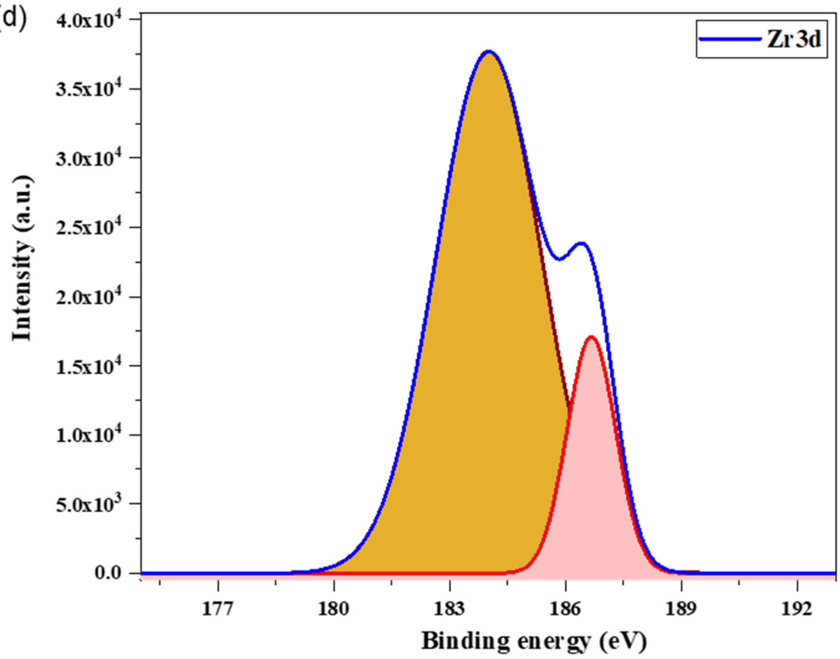
Zr3d
Intensity (a.u.)
4.0x10^4
3.5x10^4
3.0x10^4
2.5x10^4
2.0x10^4
1.5x10^4
1.0x10^4
5.0x10^3
0.0
177
180
183
186
189
192
Binding energy (eV)

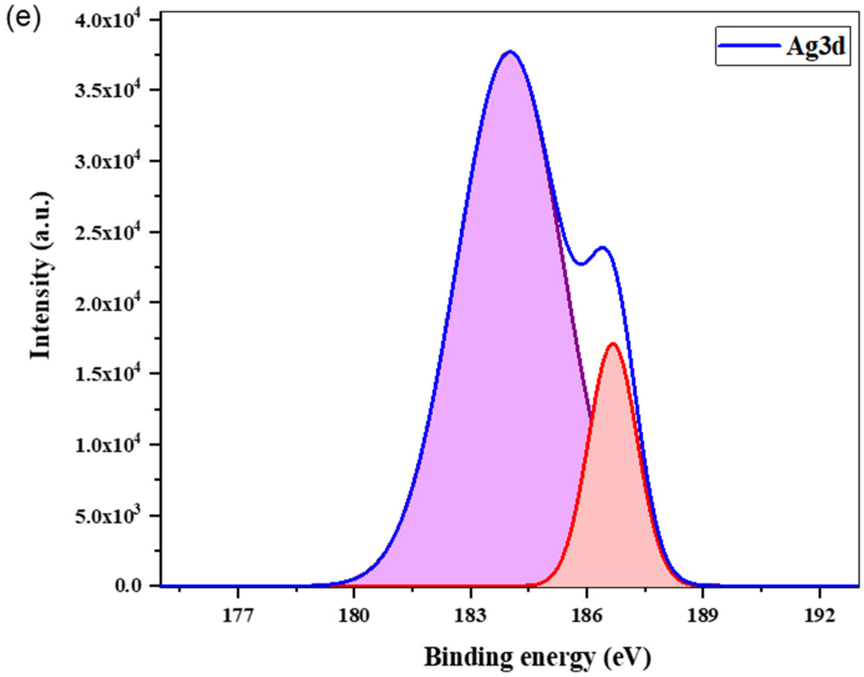

Figure S1. XPs spectra of C1s (a), Si2p (b), Zr3d (c), O1s (d) and Ag3d (e).

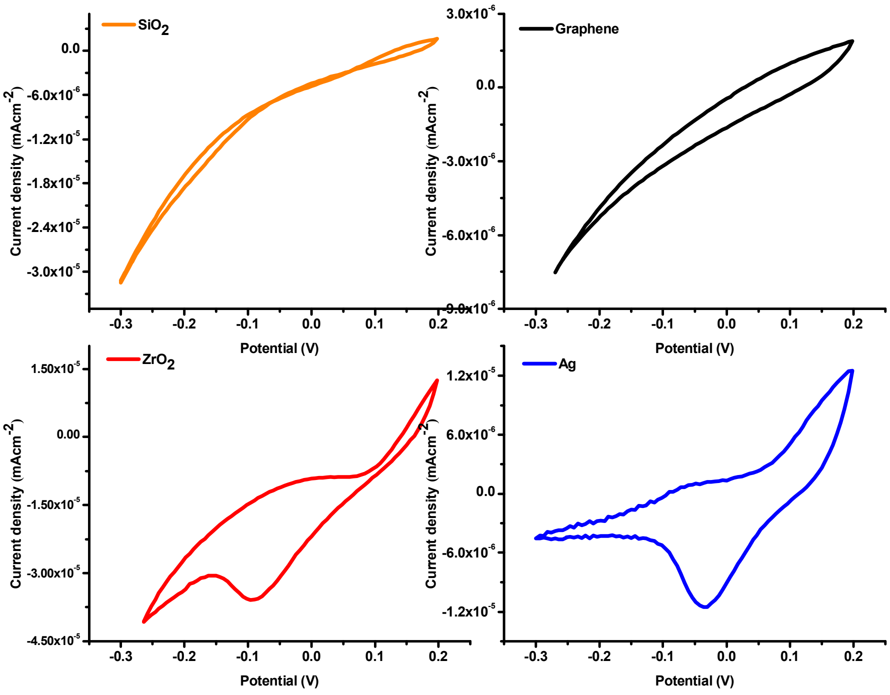

Figure S2. CV curve of SiO_2, Graphene, ZrO_2 and Ag.

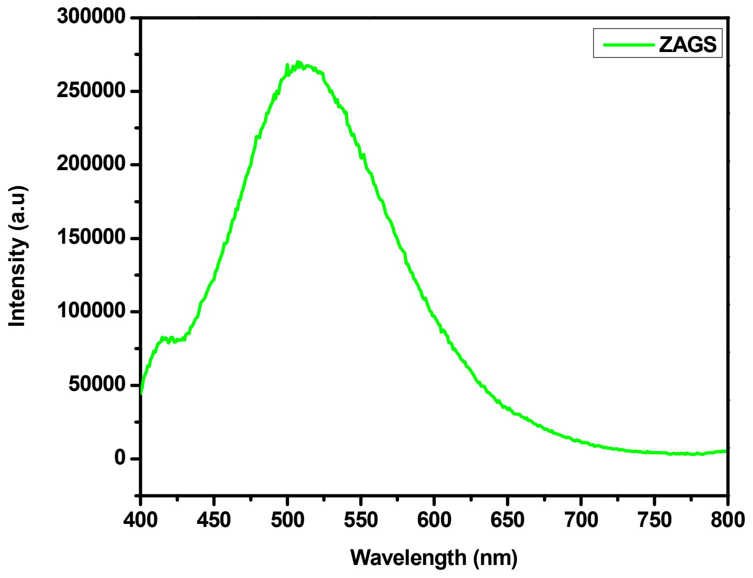

Figure S3. Pl data analysis of ZAGS.

Table S1. Comparison of glucose detection method using various sensing electrodes.

Detection method	Material	LOD (mmol/L)	Linear range (mmol/L)	Reference
Electrochemical	Ni-SnOx/PANI/CuO	0.130	1–10	[1]
Fluorimetry	Mn-doped $Zn_{0.5}Cd_{0.5}$@ZnS NRs	0.1	0.05–0.3	[2]
Colorimetry	$Cu_2(OH)_3Cl$-CeO_2 NPs-TMB	0.05	0.1–2	[3]
Electrochemical	Nafion/GOx/ZnO/FeC11SH/Au	-	0.05–1.0	[4]

References

1. Luo, J.J.; Pan, S.W.; Yang, J.H.; Chang, T.L.; Lin, P.Y.; Wu, C.L.; Liu, W.F.; Huang, X.R.; Koshevoy, I.O.; Chou, P.T.; et al. Detecting glucose levels in blood plasma and artificial tear by Au (I) Complex on the carbopol polymer: A microfluidic paper-based method. *Polymers* **2018**, *10*, 1001.

2. Tomanin, P.P.; Cherepanov, P.V.; Besford, Q.A.; Christofferson, A.J.; Amodio, A.; McConville, C.F.; Yarovsky, I.; Caruso, F.; Cavalieri, F. Cobalt phosphate nanostructures for non-enzymatic glucose sensing at physiological pH. *ACS Appl. Mater*. **2018**, *10*, 42786–42795.
3. Ahmad, R.; Ahn, M.S.; Hahn, Y.B. Fabrication of a non-enzymatic glucose sensor field-effect transistor based on vertically oriented ZnO nanorods modified with Fe_2O_3. *Electrochem. Commun.* **2017**, *77*, 107–111.
4. Zhou, Y.; Ni, X.; Ren, Z.; Ma, J.; Xu, J.; Chen, X. A flower-like NiO–SnO 2 nanocomposite and its non-enzymatic catalysis of glucose. *RSC Adv.* **2017**, *7*, 45177–45184.
